# Antigenicity is preserved with fixative solutions used in human gross anatomy: A mice brain immunohistochemistry study

**DOI:** 10.3389/fnana.2022.957358

**Published:** 2022-10-12

**Authors:** Eve-Marie Frigon, Mahsa Dadar, Denis Boire, Josefina Maranzano

**Affiliations:** ^1^Department of Anatomy, University of Quebec in Trois-Rivières, Trois-Rivières, QC, Canada; ^2^Department of Psychiatry, The Douglas Research Centre, McGill University, Montreal, QC, Canada; ^3^Department of Neurology and Neurosurgery, McConnell Brain Imaging Centre, Montreal Neurological Institute, McGill University, Montreal, QC, Canada

**Keywords:** antigenicity, fixation, histology, immunohistochemistry, human gross anatomy, mouse brain, perfusion

## Abstract

**Background:**

Histology remains the gold-standard to assess human brain biology, so *ex vivo* studies using tissue from brain banks are standard practice in neuroscientific research. However, a larger number of specimens could be obtained from gross anatomy laboratories. These specimens are fixed with solutions appropriate for dissections, but whether they also preserve brain tissue antigenicity is unclear. Therefore, we perfused mice brains with solutions used for human body preservation to assess and compare the tissue quality and antigenicity of the main cell populations.

**Materials and methods:**

Twenty-eight C57BL/6J mice were perfused with 4% formaldehyde (FAS, *N* = 9), salt-saturated solution (SSS, *N* = 9), and alcohol solution (AS, *N* = 10). The brains were cut into 40 μm sections for antigenicity analysis and were assessed by immunohistochemistry of four antigens: neuronal nuclei (NeuN), glial fibrillary acidic protein (GFAP astrocytes), ionized calcium-binding adaptor molecule 1 (Iba1-microglia), and myelin proteolipid protein (PLP). We compared the fixatives according to multiple variables: perfusion quality, ease of manipulation, tissue quality, immunohistochemistry quality, and antigenicity preservation.

**Results:**

The perfusion quality was better using FAS and worse using AS. The manipulation was very poor in SSS brains. FAS- and AS-fixed brains showed higher tissue and immunohistochemistry quality than the SSS brains. All antigens were readily observed in every specimen, regardless of the fixative solution.

**Conclusion:**

Solutions designed to preserve specimens for human gross anatomy dissections also preserve tissue antigenicity in different brain cells. This offers opportunities for the use of human brains fixed in gross anatomy laboratories to assess normal or pathological conditions.

## Introduction

Histology remains the gold-standard method to assess pathological and normal aging changes in the human brain, accurately depicting cellular morphology and the presence of specific antigens (Durand-Martel et al., 2010; Hoffmann et al., 2011; den Bakker, 2017; Pietrzak et al., 2018; Filippi et al., 2019). *In vivo* brain samples can only be obtained through biopsies, which provide small amounts of tissue and are very invasive, hence rarely performed (Quick-Weller et al., 2018). Neuroscientific histology research typically uses *ex vivo* brains obtained from brain banks, as they provide larger tissue samples, but infrequently complete brains (Vonsattel et al., 2008; Carlos et al., 2019). There are presently few active brain banks in Canada.^1^ As an alternative, the brains of bodies donated to human gross anatomy laboratories could expand the availability of tissue to the neuroscientific research community, since there are numerous body donation programs in Canada.^2^

Both brain banks and anatomy laboratories use chemical fixation to prevent the decay of the tissues (Brenner, 2014; McFadden et al., 2019). The two most widely used chemicals for tissue preservation are alcohol and formaldehyde, because of their antiseptic and antibacterial properties and their capacity to reduce autolysis (Fox et al., 1985; Brenner, 2014; Musiał et al., 2016). However, they increase the cross-linking of proteins affecting the structure of antigens (Puchtler and Meloan, 1985; Eltoum et al., 2001; Dawe et al., 2009; van Essen et al., 2010; Birkl et al., 2016; Im et al., 2019). In addition, formaldehyde distorts (Mouritzen Dam, 1979; Weisbecker, 2011) and hardens the tissue, making it less flexible (Richins et al., 1963; Tolhurst and Hart, 1990; Hayashi et al., 2014), and it is a health hazard (Fisher, 1905; Raja and Sultana, 2012; Musiał et al., 2016). Therefore, high concentrations, such as that used in brain banks and animal fixation (i.e., 4%) (Yamaguchi and Shen, 2013; McFadden et al., 2019), are avoided in human gross anatomy laboratories (Brenner, 2014).

Consequently, human anatomists have developed alternative fixative solutions that combine various chemicals, allowing lower concentrations of formaldehyde. For example, a salt-saturated solution preserves the body flexibility, optimizing dissection procedures (Coleman and Kogan, 1998; Hayashi et al., 2014). Another example is a solution of multiple alcohols tailored to perform neurosurgical simulations, since the density and retraction properties of the tissue remain closer to the *in vivo* brain (Benet et al., 2014). Despite the good tissue preservation and dissection properties provided by salt and alcohol solutions, it is unclear whether they preserve cellular morphology and antigenicity of the main brain cell populations (Lyck et al., 2008; Benet et al., 2014).

Finally, in gross anatomy laboratories, the fixation/embalming procedures are performed by arterial perfusion within 48 h following death (Frølich et al., 1984; Nicholson et al., 2005; Brenner, 2014; Maranzano et al., 2020). This inconstant post-mortem delay introduces a variable degree of clotting, which combined with vascular stenosis may negatively impact the perfusion, confounding the fixation quality (Turkoglu et al., 2014). Moreover, human brains may take up to 6 months to be fully fixed, which is an important delay to consider when designing human studies (Dawe et al., 2009; Raman et al., 2017).

In this study, we avoided the post-mortem, and fixation delays by using mice brains, which are fixed immediately by transcardial perfusion of the deeply anesthetized animals. We then assessed the overall histology procedures and quality (i.e., perfusion quality, ease of manipulation of the slices, and tissue quality), as well as antigenicity preservation (assessed by immunohistochemistry of four antigens) using three solutions. We also performed (in a subgroup of animals) an assessment of immunofluorescence (IF), to determine its feasibility, since it is another frequently used staining technique that allows simultaneous visualization of several target antigens. Since 4% formaldehyde (FAS) is the fixative of choice of brain banks and animal fixation protocols, we hypothesized that the histology quality and antigenicity would be superior in mice brains fixed with FAS than those fixed with a salt-saturated solution (SSS) or an alcohol solution (AS) used in human gross anatomy (Coleman and Kogan, 1998; van Essen et al., 2010; Benet et al., 2014; McFadden et al., 2019).

## Materials and methods

### Population

We used 28 6-month-old C57BL/6J mice (14 females and 14 males) (Table 1) raised in an enriched environment with controlled ventilation (45–60% humidity) and temperature (20–25°C) on a 12-h daylight schedule. We followed the guidelines of the Canadian Council on Animal Care, and all procedures were approved by the Ethics Committee of the Université du Québec à Trois-Rivières.

**TABLE 1:** Mice data.

Fixative	Sex	Age (days)	Weight (g)
FAS	F	182	28.1
		182	28.2
		182	32.3
		187	26.5
		200	29.3
	M	182	33.7
		214	26.3
		214	28.4
		220	39.1
SSS	F	182	27.8
		182	27.7
		187	27.5
		200	31.1
	M	182	29.7
		183	27.7
		189	25.2
		215	27.4
		220	34.5
AS	F	182	29.6
		182	29.3
		187	26.9
		200	25.9
		200	22.9
	M	167	33.6
		182	31.6
		183	29.5
		215	27.0
		215	35.4

M, male; F, female; FAS, formaldehyde solution; SSS, salt-saturated solution; AS, alcohol solution.

### Fixation procedure

Mice were sacrificed by transcardiac perfusion with phosphate-buffered saline (PBS) (0.1 M; 0.9% NaCl), followed by a 5-min injection of one of the following solutions (randomly assigned): (1) FAS (*N* = 9), (2) SSS (*N* = 9), and (3) AS (*N* = 10) (Table 2). The perfusion was performed using a gravity technique (Paul et al., 2008; Au-Rana et al., 2022), with the same caliber tubes (15 drops/ml) and cannulas. FAS and SSS bags were placed at the same level, obtaining a similar continuous fluid flow. Due to the higher viscosity and density of the AS, owing to its greater concentration of glycerol, the AS bag was placed 50 cm higher to obtain a similar continuous flow than that of the other solutions. We then extracted and assessed the brain color, which indicates the amount of remaining blood, to determine the post-fixation time by immersion in the same solution for each brain (2 h for pink brains, 1.5 h for heterogeneous color brains, and 1 h for beige brains). We then immersed the brains in 30% sucrose in 0.1 M PBS for cryoprotection overnight. Finally, the specimens were frozen in dry ice and stored at −80°C for a short period (1 day to 2 weeks) until use.

**TABLE 2:** Fixative’s components.

FAS	SSS (Coleman and Kogan, 1998)	AS (Benet et al., 2014)
4% formaldehyde	0.296% formaldehyde	0.851% formaldehyde
0.1 M PBS	36% NaCl	62.4% ethanol
	0.72% phenol	10.2% phenol
	2% glycerol	17% glycerol
	16% isopropylic alcohol	

### Histology processing

Brains were cut using a cryostat (Leica CM1950) in 40-μm coronal sections at −19°C and rinsed three times for 5 min in 0.1 M PBS. We then incubated the floating sections for 30 min in an aqueous solution of 20% methanol and 0.5% H_2_O_2_ to quench endogenous peroxidase. Sections were rinsed again three times for 5 min in 0.1 M PBS before a 2-h incubation at room temperature in blocking solution (3% Normal Donkey Serum; 0.5% Bovine Serum Albumin; 0.3% Triton X-100 in 0.1 M PBS). Then, we incubated the sections overnight at 4°C in the same blocking solution with primary antibodies (Table 3). We rinsed again three times for 5 min in 0.1 M PBS before incubation of the sections for 2 h in donkey anti-rabbit biotinylated secondary antibody (1:500) at room temperature in the same blocking solution. Sections were rinsed again (3 × 5 min in 0.1 M PBS) and incubated in an avidin-biotin complex (ABC) kit for 30 min in the dark (Vector Laboratories, Newar, CA USA, catalog#VECTPK6100). We rinsed three times for 5 min in TRIS-buffered saline (TBS) (0.05 M; 0.9% NaCl) before the final incubation in 0.07% diaminobenzidine (DAB) (MilliporeSigma, Burlington, MA, USA, catalog #D5905-50TAB) with 0.024% H_2_O_2_ in TBS for 10 min. We rinsed the slices in 0.01 M phosphate-buffered (PB) before mounting on 2% gelatin-subbed slides. Finally, sections were dehydrated in graded alcohol (5 min each: 70% ethanol, 95% ethanol, 2X 100% ethanol, and 2X xylene) and coverslipped using Eukitt.

**TABLE 3:** Immunohistochemistry antibodies.

Primary antibodies	Animal	Laboratory	Dilution	Code
Anti-neuronal nuclei (NeuN)	Rabbit	Abcam	1:3,000	Ab177487
Anti-glial fibrillary acidic protein (GFAP)	Rabbit		1:1,000	Ab68428
Anti-ionized calcium-binding adaptor molecule1 (Iba1)	Rabbit		1:1,000	Ab178847
Anti-myelin proteolipid protein (PLP)	Rabbit		1:3,000	Ab254363

**Secondary antibody**	**Animal**	**Laboratory**	**Dilution**	**Code**

Anti-rabbit biotinylated	Donkey	NovusBio	1:500	NBP1-75274

### Variables of interest

We qualitatively assessed five variables: 1—perfusion quality, 2—ease of manipulation, 3—tissue quality, 4—antigenicity preservation, and 5—immunohistochemistry quality. They were evaluated according to different criteria, where the highest score reflected the highest quality of the element.

#### Perfusion quality

We used three criteria to assess this variable, namely the presence or absence of signs of perfusion through the right ventricle, the brain color, and the presence or absence of dilated vessels.

##### Signs of perfusion through the right ventricle

When the fixative goes through the right ventricle, it will result in a poorer perfusion since the fixative goes into the pulmonary circulation and indirectly to the brain instead of accessing the systemic (left) circulation, which goes directly to the brain. This was assessed since the higher the density of a solution (namely the AS with a high concentration of glycerol), the greater the potential for damage of the thin interventricular septum, with the unintentional passage of the solution from the left to the right ventricle. The indicative signs of perfusion in the right ventricle, used for the score, were red liver, white lungs, and fluid flowing through the nose.

Scores: 3 signs = 0, 2 signs = 1, 1 sign = 2, and 0 sign = 3.

##### Brain color

The brain color is an indication of the quality of perfusion, whereas the pink color indicates remaining blood in the brain, showing that the perfusion was less effective. In these cases, brains were post-fixed for a longer period to better our odds of a good histology.

**FIGURE 1:**
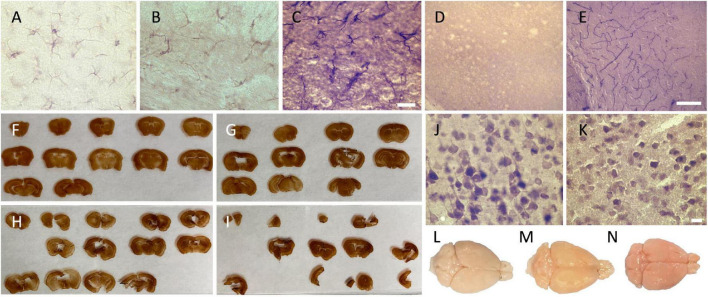
Photomicrographs of the different categorical variables that were assessed. Photomicrographs (60X) of the background intensity **(A)** light = 2; **(B)** intermediate = 1; and **(C)** dark = 0, acquired in the external capsule of GFAP-labeled slices, where we can see the presence of labeled astrocytes. Scale bar = 20 μm [valid for **(A–C)**]. **(D)** Photomicrographs of the presence of dilated vessels (20X) (perfusion quality) and **(E)** of endogenous peroxidase presence in the blood vessels (red cells and endothelial cells) (20X) (histology quality) in the cortex, also acquired in GFAP-labeled slices (no astrocyte on the picture since they were only present in the white matter). Scale bar = 100 μm [valid for **(D,E)**]. Ease of manipulation **(F)** very good = 3; **(G)** good = 2; **(H)** poor = 1; and **(I)** very poor = 0. Photomicrographs (60X) of **(J)** a uniform neuropil and regular cell contours in the secondary motor cortex, and **(K)** a fissured and irregular neuropil and cell contours, also acquired in the secondary motor cortex of the closest slice to figure 33 in the Mouse Brain Atlas. Scale bar = 10 μm [valid for **(J,K)**]. Brain color (perfusion quality) **(L)** beige = 2; **(M)** heterogenous = 1; and **(N)** pink = 0.

Scores: pink = 0 (Figure 1N), heterogenous = 1 (Figure 1M), and beige = 2 (Figure 1L).

##### Dilated vessels

A higher injection pressure during the perfusion made the vessels burst, negatively impacting the subsequent microscopic analysis, since the antigens may be masked out by these dilated vessels. This was assessed in the NeuN sections of the specimens.

Scores: presence = 0 (Figure 1D) and absence = 1.

#### Ease of manipulation

We used four signs to assess this variable that were equally assessed with scores from 0 to 1 and then summed up to obtain one variable with a possible score between 0 and 4. The criteria were namely 1—slice consistency (soft = 0 or firm = 1), 2—tears (presence = 0 or absence = 1), 3—slices on the brush (sticking = 0 or not adherent = 1), and 4—rolling slices (presence = 0 or absence = 1).

Scores: very poor (4 signs present = 0) (Figure 1I), poor (3 signs present = 1) (Figure 1H), good (1 or 2 signs present = 2) (Figure 1G), or very good (no sign present = 3) (Figure 1F).

#### Tissue quality

We used three criteria to assess this variable, namely the neuropil quality, the cellular shape, and the presence or absence of peroxidase-labeled vessels.

##### Neuropil quality

A fissured neuropil reflects the degradation of the tissue. This was assessed in the NeuN sections of the specimens.

Scores: fissured = 0 (Figure 1K) and uniform = 1 (Figure 1J).

##### Cell shape

Shrunken and shriveled contours (irregular) are signs of dehydration of the neurons. This was assessed in the NeuN sections of the specimens, in the second and third layers of the secondary motor cortex. The score reflected the characteristic of all the cells in this region of interest.

Scores: irregular (shrunken/shriveled) = 0 (Figure 1K) and regular (smooth) = 1 (Figure 1J).

##### Endogenous peroxidase presence in blood vessels

The presence of endogenous peroxidase in the endothelial and red cells in the blood vessels (represented as dark brown lines, Figure 1E) shows that the quenching procedure was not sufficient for the specimen. This criterion was assessed because it may negatively impact the subsequent microscopic analysis by masking out the antigens. This was assessed in the GFAP sections of the specimens, since they were easily visible because of the absence of cells in the cortex (astrocytes were labeled in the white matter only).

Scores: presence = 0 (Figure 1E) and absence = 1.

#### Antigenicity preservation

We looked at slices targeted for all the antigens to verify the labeling. No labeling would show the destruction and/or too much cross-linking of this specific antigen by the fixative. This was assessed for every antigen.

Scores: absence = 0 and presence = 1.

#### Immunohistochemistry quality

We used two criteria to assess this variable, namely the degree of antibody penetration in the thickness of the slices and the darkness of the tissue background.

##### Antibody penetration

This was assessed by looking through the full thickness of the slices. The antibody penetration shows the permeability of the tissue. The incomplete condition means the middle of the slice was not labeled, and the complete depicts full penetration at all depth levels. This was assessed for every antigen.

Scores: incomplete = 0 and complete = 1.

##### Tissue background

The background reflects the quality of the chemical reaction of the DAB labeling. This was assessed for every antigen.

Scores: dark = 0 (Figure 1C), intermediate = 1 (Figure 1B), and light = 2 (Figure 1A).

### Microscopy

We assessed the tissue quality, the antigenicity preservation, and the immunohistochemistry quality using a bright-field microscope (Olympus, Tokyo, Japan, BX51W1) controlled by Neurolucida software (MBF Bioscience, Williston, VT, USA). We graded these variables by looking through the full thickness of sections immunolabeled for the four antigens with 60X objective (60X, UPlanSApo 60x/1.40 Oil∞/0.17/FN26.5 UIS2). First, we performed a quality control of all stained slices of each brain, to ensure that all antigens were present and homogeneously distributed in all available slices. Then, we chose to assess the slice stained for the antigen of interest that was the closest to figure 33 of the Mouse Brain Atlas (0.22 mm posterior to Bregma) (Paxinos, 2001), since it is a level of the brain easy to identify that was of sufficient quality to be assessed across specimens. We then visually assessed the variables by scanning the secondary motor cortex of these slices because it is a cortical region that is unambiguous to delineate according to neuronal morphology (Caviness, 1975). For GFAP-stained slices, we visually assessed the cells in the external capsule, since there were no astrocytes in the secondary motor cortex gray matter.

### Statistical analyses

We compared the categorical variables across the three fixative groups using chi-square tests. All the statistical analyses were corrected for multiple comparisons using Bonferroni correction and were performed using SPSS statistics software (28.0.0 version).

### Immunofluorescence staining

#### Histology processing

In a subgroup of *N* = 9 mice (FAS = 3, SSS = 3, and AS = 3), we used two free-floating slices (the remaining sections closest to the Figure 33 of the Mouse Brain Atlas) that were rinsed three times for 5 min in 0.1 M PBS. We then incubated the floating sections 20 min in an aqueous solution of 20% methanol. We rinsed again before a 2-h incubation at room temperature in blocking solution (2% Normal Donkey Serum; 0.75% Glycine and 0.3% Triton X-100 in 0.1 M PBS). We then incubated the sections in the same blocking solution with primary antibodies for double staining of neuronal nuclei (NeuN) and astrocytes (GFAP) (Table 4). We rinsed again three times for 5 min in 0.1 M PBS before incubation in secondary antibodies (in the same blocking solution) for 2 h (Table 4). Finally, sections were rinsed before mounting on 2% gelatin-subbed slides and coverslipped using Mowiol mounting medium.

**TABLE 4:** Immunofluorescence antibodies.

Primary antibodies	Animal	Laboratory	Dilution	Code
Anti-neuronal nuclei (NeuN)	Guinea pig	Millipore sigma	1:6,000	ABN90
Anti-glial fibrillary acidic protein (GFAP)	Rabbit	Abcam	1:1,000	Ab68428

**Secondary antibody**	**Animal**	**Laboratory**	**Dilution**	**Code**

Anti-rabbit alexa fluor 555	Donkey	BioLegends	1:500	406412
Anti-guinea pig alexa fluor 488		Jackson laboratories	1:500	706-545-158

#### Microscopy and analysis

We used a DSU spinning disk microscope (Olympus, Tokyo, Japan, BX51W1) (60X objective UPlanSApo 60x/1.40 Oil∞/0.17/FN26.5 UIS2) to qualitatively assess, in a subgroup of nine mice, the immunofluorescence staining quality (IF). We visually inspected three criteria: 1—the antigenicity preservation, 2—the auto-fluorescence, and 3—the tissue quality. No statistical analysis was applied on these results since we had no statistical power when using *N* = 3 mice per group.

##### Antigenicity preservation

Antigenicity preservation was assessed by the presence or absence of both labeled antigens (neurons and astrocytes) in all the immunolabeled slices of all specimens.

##### Auto-fluorescence

This criterion was assessed by observing the presence or absence of fluorescent artifacts in the secondary motor cortex of the negative control-stained slices (same IF protocol but incubated without primary antibodies). We qualitatively and visually assessed the auto-fluorescence by looking through the backgrounds of NeuN and GFAP-stained slices.

##### Tissue quality

The tissue quality was visually assessed by imaging with differential interferential contrast illumination (DIC) of the fluorescent control slices, showing the neuropil and cellular texture. We also assessed the cellular shape and the antibody penetration in the Alexa-488 for NeuN channel and Alexa-555 for GFAP channel, respectively.

## Results

### Perfusion quality

**FIGURE 2:**
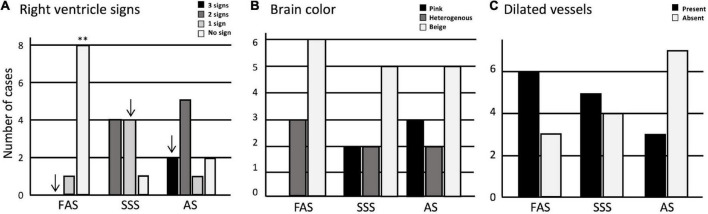
Bar charts of the perfusion quality. **(A)** Signs of perfusion in the right ventricle; **(B)** brain color; **(C)** presence or absence of dilated vessels. Significance after Bonferroni correction: ^**^p < 0.001; _↓_ = significant before Bonferroni correction; FAS, formaldehyde solution; SSS, salt-saturated solution; AS, alcohol solution.

We started by assessing the perfusion quality, finding a significantly higher number of cases without signs of perfusion through the right ventricle in the FAS-fixed specimens (*p* = 0.0002) (Figure 2A). The SSS and AS show a higher number of cases with one sign and three signs of perfusion in the right ventricle, respectively, but this observation did not retain significance after Bonferroni correction. We did not find significant differences in brain color, nor dilated vessels across the three fixatives (Figures 2B,C). However, pink-colored brains were only observed when fixed with AS (*N* = 3) and SSS (*N* = 2). Also, although not statistically significant, there were fewer AS specimens showing dilated vessels.

### Ease of manipulation

**FIGURE 3:**
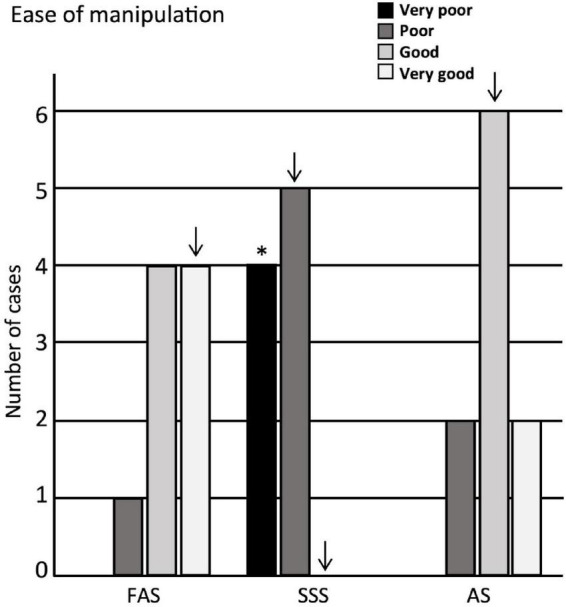
Bar charts of the ease of manipulation. Very poor = 4 signs; poor = 3 signs; good = 1 or 2 signs; very good = no sign. Significance after Bonferroni correction: *p < 0.05; _↓_ = significant before Bonferroni correction; FAS, formaldehyde solution; SSS, salt-saturated solution; AS, alcohol solution.

We then assessed the ease of manipulation, finding that the scores obtained for the brains fixed with SSS were significantly lower (score = very poor) (*p* = 0.002) than those of the brains treated with the other two solutions (Figure 3). We also found that the scores of the manipulations corresponded more often to a poor manipulation of SSS slices, to a very good manipulation of FAS slices, while the AS had good scores, but these observations did not retain significance after Bonferroni correction.

### Tissue quality

**FIGURE 4:**
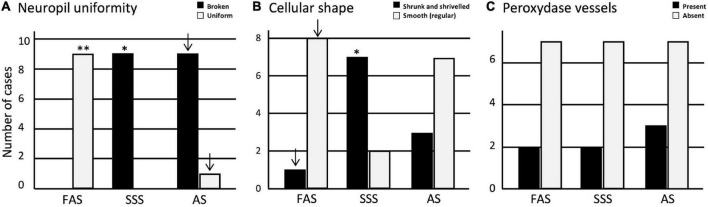
Bar charts of the tissue quality. **(A)** Neuropil uniformity; **(B)** cellular shape; **(C)** presence or absence of peroxidase vessels. Significance after Bonferroni correction, *p < 0.05, ^**^p < 0.001; _↓_ = significant before Bonferroni correction; FAS, formaldehyde solution; SSS, salt-saturated solution; AS, alcohol solution.

Regarding the tissue quality, we found that the neuropil was significantly more often uniform in FAS-fixed specimens (*p* < 0.001). It was significantly more often fissured in the SSS-fixed brains (*p* = 0.007); AS-fixed brains also showed more specimens with a fissured neuropil, which did not retain significance after Bonferroni correction (Figure 4A). Regarding the cellular shape, the specimens fixed with SSS showed significantly more often irregular cells (*p* = 0.004) compared to the brains fixed with AS or FAS (Figure 4B). The FAS-fixed specimens showed mostly regular cells, but this did not retain significance after Bonferroni correction. Finally, we found no significant differences for the occurrence of peroxidase-labeled vessels across the three fixatives (Figure 4C).

### Antigenicity preservation

**FIGURE 5:**
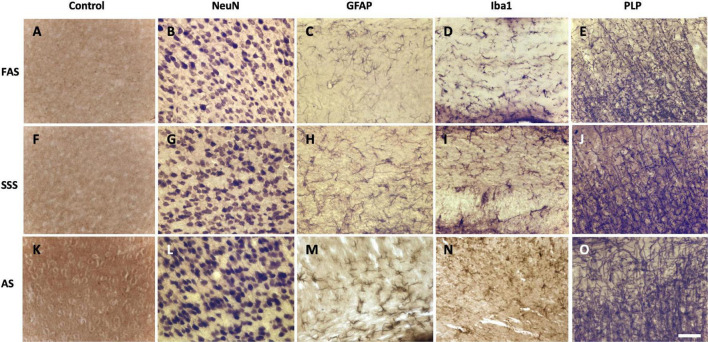
Photomicrographs (40X) of the different antigens of brain samples fixed with three different solutions. **(A,F,K)** Negative control sections, **(B,G,L)** neuronal cell bodies (NeuN), **(C,H,M)** astrocytes (GFAP), **(D,I,N)** microglia (Iba1), and **(E,J,O)** myelin fibers (PLP) fixed with FAS, SSS, and AS, respectively. Photomicrographs were acquired in layers 2–3 of the secondary motor cortex of sections from 0.04 to 0.46 mm posterior to Bregma (the Mouse Brain Atlas). Photomicrographs were acquired in similar background specimens (light for NeuN and Iba1, intermediate for GFAP and PLP) to increase the visual comparability of the antigens, by avoiding any dark backgrounds. Scale bar = 100 μm.

Negative control sections (Figures 5A,F,K) were compared to the immunolabeled sections that show that the three fixatives preserved antigenicity for the four antigens of interest. Neurons (NeuN) were labeled and showed the neuronal cell bodies (Figures 5B,G,L). Astrocytes (GFAP) (Figures 5C,H,M) and microglia cells (Iba1) (Figures 5D,I,N) clearly showed cell bodies and fine cellular processes. Astrocytes were present in the white matter tracts only. Finally, PLP clearly labeled myelinated fibers (Figures 5E,J,O) in all the specimens. The four tested antigens were present and comparable in every mouse, and therefore, there were no differences between the three fixatives.

### Immunohistochemistry quality

**FIGURE 6:**
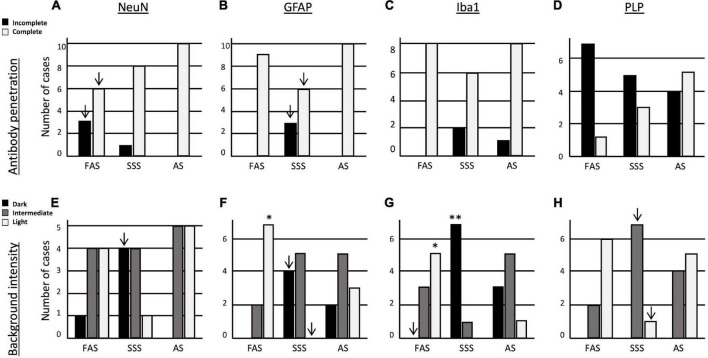
Bar charts of the immunolabeling quality. Antibody penetration of **(A)** NeuN, **(B)** GFAP, **(C)** Iba1, and **(D)** PLP. Tissue background of **(E)** NeuN, **(F)** GFAP, **(G)** Iba1, and **(H)** PLP. Significance after Bonferroni correction: *p < 0.05, ^**^p < 0.001; _↓_ = significant before Bonferroni correction; FAS, formaldehyde solution; SSS, salt-saturated solution; AS, alcohol solution.

Since all the antigens were present, we were able to assess the quality of immunolabeling using two criteria. First, we assessed the antibody penetration in the depth of the sections for the four antigens. NeuN penetrated completely in all the specimens fixed with AS, while FAS-fixed brains showed three specimens and SSS one specimen with incomplete penetration (Figure 6A). GFAP penetrated completely in the FAS- and AS-fixed brains, while three SSS specimens showed incomplete penetration (Figure 6B). These results were statistically significant only before Bonferroni correction. We did not find any differences in the penetration of Iba1 nor PLP between the three fixatives (Figure 6C). However, PLP showed the poorest antibody penetration, especially in FAS-fixed brains (seven incomplete penetration), with AS-fixed brains showing the best PLP antibody penetration (five complete penetrations) although this was not statistically different across the three solutions (Figure 6D).

Finally, we assessed the background intensity in slices labeled with the four antigens. The NeuN background labeling was more often darker in brains fixed with SSS, but it did not retain significance after Bonferroni correction (Figure 6E). The GFAP background was significantly more often lighter in brains fixed with FAS than the two other solutions (*p* = 0.001). In addition, SSS-fixed brains showed a higher occurrence of dark backgrounds and absence of light backgrounds of GFAP than the two other fixatives, but not retaining significance after Bonferroni correction (Figure 6F). In Iba1-labeled slices, we found significantly more often dark backgrounds in the specimens fixed with SSS (*p* = 0.0009), while more light backgrounds were present in FAS-fixed brains (*p* = 0.002). We also never observed dark backgrounds in the specimens fixed with FAS, but this did not retain significance after Bonferroni correction (Figure 6G). In the PLP-labeled slices, we found no dark backgrounds for any of the specimens. The SSS-fixed brains present more often an intermediate background and less often a light background than the two other solutions, but this did not retain significance after Bonferroni correction (Figure 6H).

### Immunofluorescence staining

#### Antigenicity preservation

**FIGURE 7:**
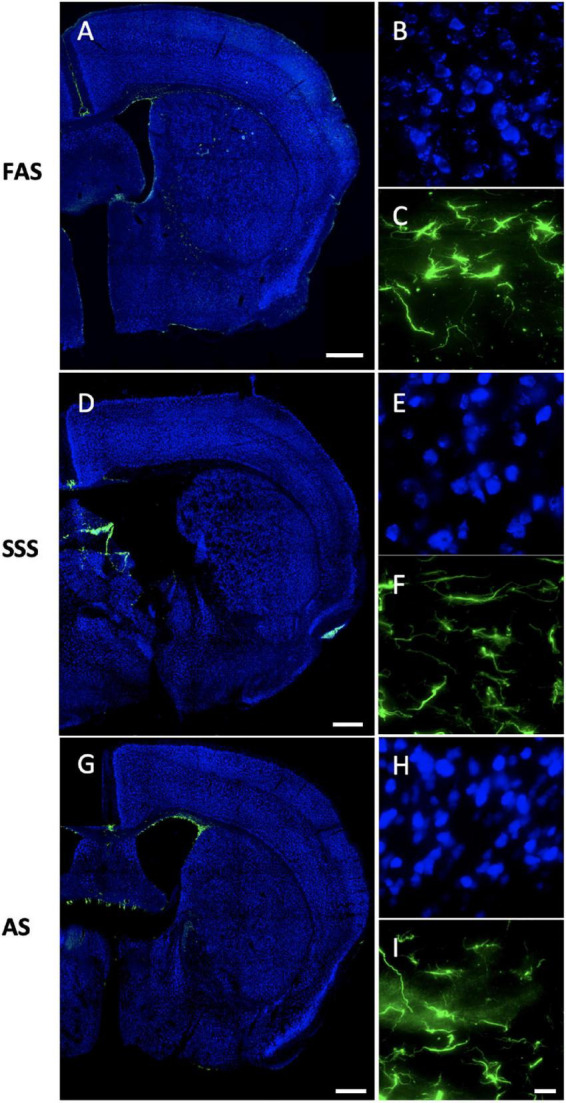
Immunofluorescence antigenicity. Slide scans (10X) (the remaining closest slices of figure 33 (0.22 mm posterior to bregma of the Mouse Brain Atlas, Paxinos, 2001) of double immunostaining of NeuN (Alexa-488 showed in fake color blue) and GFAP (Alexa-555 showed in fake color green) labeled in slices of **(A)** FAS-fixed brain, **(D)** SSS-fixed brain, and **(G)** AS-fixed brain. Scale bars = 500 μm. Photomicrographs (60X) of NeuN in the layers 2–3 of the secondary motor cortex (blue) for **(B)** FAS, **(E)** SSS, and **(H)** AS-fixed brains. Photomicrographs (60X) of GFAP in the external capsule (green) for **(C)** FAS, **(F)** SSS, and **(I)** AS. Scale bar = 15 μm [valid for **(B,C,E,F,H, I)**].

Regarding the IF staining quality of the chosen antigens, we first found that both antigens (NeuN and GFAP) were present in all stained slices in the nine mice and across the three fixatives. The labeling was homogenous for NeuN (found in the full slices), and astrocytes (GFAP) were present in the white matter and the striatum, no matter the fixative (Figure 7).

#### Auto-fluorescence

We found that AS-fixed brains showed a little more auto-fluorescence in the 555 channel (Figure 7I) than the brains fixed with the two other solutions. Also, FAS-fixed brains showed higher auto-fluorescence in the 488 channel (Figure 7B) (visible granules in the NeuN background). However, it appeared to be at a comparable level of auto-fluorescence in all the channels (Figure 7).

#### Tissue quality

**FIGURE 8:**
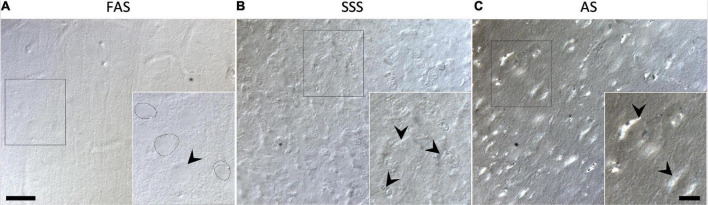
Tissue integrity of the immunofluorescence processed slices. Photomicrographs (60X) of DIC illuminated tissue slices of the immunofluorescence negative control slices showing the texture of the brain tissue when fixed with **(A)** FAS, **(B)** SSS, and **(C)** AS. Insert in A shows regular cell contours and smooth neuropil. Black arrowheads in insert **(B)** show shrunken and shriveled cells, while showing a broken neuropil in insert **(C)**. Left scale bar = 25 μm [valid for **(A–C)**]. Right scale bar = 10 μm (valid for all three inserts).

Neurons labeled using IF were irregular (shrunken and shriveled) when fixed with SSS (Figure 7E). The antibody penetration was complete through the slice thickness in all slices of the nine specimens, no matter the fixative. Figure 8 also shows the neuropil and cell texture of the tissue imaged with DIC optics in brains fixed with FAS, SSS, and AS. FAS-fixed slices show a smooth texture, round-shape cells with few artifacts (Figure 8A), while the SSS slice shows shrunken and shriveled cells (Figure 8B, black arrowheads). The AS-fixed brains show broken neuropil, since the contours of the cells are well delineated (Figure 8C, arrowheads).

## Discussion

In this study, we investigated the impact of three different fixative solutions, one routinely used in brain banks and animal fixation studies, as well as two solutions used in human gross anatomy laboratories, on the histological quality of brain tissue immunostained for common antigens used in neuroscience research. Our goal was to determine whether the fixatives used in human anatomy procedures could produce histological sections of sufficient quality and preserve antigenicity when administered by perfusion without the confounding effect of post-mortem delay.

### Perfusion quality

We first assessed the perfusion quality since it is the process by which the fixative is delivered to the organ, and potential differences in the flow through certain vessels could compromise the quality of the fixation. Specifically, we foresaw that the AS might be more difficult to perfuse because it contains a high concentration of glycerol (17%) (Benet et al., 2014), which has a higher density and viscosity (Takamura et al., 2012; Ferreira et al., 2017). This caused the thin interventricular septum of the mouse to be broken more often, decreasing the perfusion quality of the AS specimens, which showed a higher frequency of signs of perfusion in the right ventricle, although not statistically significant. As expected, brains were easier to perfuse with FAS, showing significantly fewer signs of right ventricle injection (Winkelman and Beenackers, 2000).

Also, as anticipated, the AS-fixed brains were more often pink, reflecting a higher amount of blood in the brain after perfusion, hence a jeopardized perfusion (note that we did not exclude any of the pink-colored specimens; instead, we treated them with longer post-fixation times in the corresponding fixative solution and proceeded to assess the histology variables). Finally, we observed that AS-fixed brains were less prone to showing dilated vessels, which was an unexpected finding considering that the perfusion was done using a higher bag placement of a higher density solution, hence a higher pressure. We speculate that not the density, but the higher viscosity, which determines a slower and weaker flow through the brain vessels, is responsible for the less frequent occurrence of dilated vessels (Takamura et al., 2012; Ferreira et al., 2017; Wilkes, 2017). Alternatively, since dilated vessels may arise as a freezing artifact, the less frequent occurrence in AS specimens could be related to a protective effect against this freezing artifact determined by alcohol, namely glycerol (Bhattacharya, 2018; Kar et al., 2019; Zhang et al., 2020).

### Ease of manipulation

We then assessed the ease of manipulation, which determines the ability to cut and mount a full slice, required for an accurate histology analysis. Softer tissue is more fragile, hence very difficult to cut into a full slice with a cryostat. Also, viscous tissue sticks to the brush used to manipulate the slices, which enhances tears or loss of tissue. In this regard, we found that the brains fixed with SSS were the most difficult to manipulate and the FAS-fixed slices were the easiest. This is in agreement with studies on human brains showing that FAS increases the rigidity of the tissue, while SSS-fixed brains are softer (Fox et al., 1985; Coleman and Kogan, 1998; Kiernan, 2000; Eltoum et al., 2001; Nicholson et al., 2005; Weisbecker, 2011; Brenner, 2014; Hayashi et al., 2014; Balta et al., 2015; Musiał et al., 2016; Haizuka et al., 2018; McFadden et al., 2019). Fortunately, even in the brains that were very soft, and most difficult to manipulate, we could still obtain analyzable slices for all the mice. Moreover, we found that the manipulation of AS-fixed brains was mostly good, and this means the fixative is sufficiently effective to produce tissue with a texture suitable for histology manipulation despite the poorer perfusion results that we obtained.

### Tissue quality

Regarding the tissue quality, we assessed the neuropil and cellular shape since these characteristics reflect the changes in cell morphology that may be impacted by the different chemicals used for fixation (Troiano et al., 2009; Spencer and Bancroft, 2017). We speculate that the shrunken aspect of the neuronal cell bodies observed in SSS brains, but not in the FAS nor AS-fixed specimens, is related to the high concentration of isopropyl alcohol, which is a potent dehydrating fluid, only present in the SSS (Viktorov and Proshin, 2003). In addition, isopropyl alcohol could have also affected the higher occurrence of fissured neuropil in these brains. In the AS-fixed brains, the higher frequency of fissured neuropil may be due to the high concentration of ethanol, also used as a dehydrating agent (Viktorov and Proshin, 2003). However, despite the poorer tissue quality of AS- and SSS-fixed brains, all specimens clearly showed the targeted cells (Figure 5).

### Antigenicity preservation

In addition to morphological tissue characteristics, we were mostly interested in assessing the presence or absence of antigens in the brains. The detection and quantification of antigens present in the main cells of the brain are essential in a wide range of topics of neuroscientific research, from demyelinating diseases to neurodegenerative processes (Durand-Martel et al., 2010; Hoffmann et al., 2011; Pietrzak et al., 2018; Filippi et al., 2019). We found that neurons were homogeneously labeled by NeuN, and myelinated axons by PLP. However, astrocytes were only present in the white matter tracts and surrounding the ventricles, while absent in the cortical gray matter. This is commensurate with multiple studies that characterize the two types of astrocytes: 1—fibrous, mostly present in the white matter tracts, and 2—protoplasmic, uniformly distributed through the cortical gray matter (Privat et al., 1995; Chen and Swanson, 2003; Sun and Jakobs, 2012; Khakh and Deneen, 2019). Indeed, our results showed that GFAP is only expressed in the fibrous and radial populations of astrocytes, since there were no protoplasmic astrocytes labeled in the cortex, also in accordance with other studies showing that GFAP is most specific to fibrous astrocytes, and that protoplasmic astrocytes do not (or less) express GFAP (Walz, 2000; Chen and Swanson, 2003; Sun and Jakobs, 2012). Finally, microglia were labeled and homogeneously distributed throughout the slices, since Iba1 is commonly used and known to be expressed in both microglial and macrophage cells (Ito et al., 1998; Stankov et al., 2015; Hendrickx et al., 2017). Overall, our results showed that antigenicity for all the targets was equally preserved in brains fixed with any solution (Figure 5), opening the door to the use of brains fixed with solutions currently reserved for gross anatomy purposes.

### Immunohistochemistry quality

Finally, we assessed two aspects of the immunolabeling quality that may impact the antigen detection. First, the background labeling, since the darker this background, the lower the contrast with the cells of interest that we aim to visualize, potentially, under-detecting them. Our work showed that SSS brains had the darkest backgrounds, but this did not preclude, in any specimens, the identification of the targeted antigens. Also, specimens fixed with the other two solutions also showed, albeit with a lower frequency, a dark background in some specimens. This reflects that backgrounds are not systematically always bright, intermediate, or dark for a given antigen or a given fixative solution, but this does not prevent an accurate antigen visualization. Second, we assessed the penetration of the antibodies in the full thickness of the slice, which will determine the visualization of the cells at any depth level. This penetration represents the permeabilization of the tissues, differently modified according to the chemical treatment. We found that the AS-fixed brains consistently showed better penetration for all antigens, including the suboptimal PLP, which was relatively less suboptimal with AS than with SSS and FAS. We speculate that this could be related to the chemical mixture of the AS, which contains ethanol (resemblance of methanol), an alcohol known to permeabilize the membranes (Yuan et al., 2017) (Cell signaling Technology, Danvers, MA, USA and Novus Biologicals, Centennial, CO, USA).^3, 4^

### Immunofluorescence staining

The antigenicity preservation was the same for NeuN and GFAP using IF and IHC, since all the antigens were present in all slices of all specimens. Neurons were labeled homogeneously through the cortex and deep gray matter, while astrocytes were present homogeneously in the white matter (internal and external capsules). Regarding the auto-fluorescence criterion, it was present in the two channels (488 and 555) of all specimens, while a little higher in NeuN for FAS-fixed brains and in 555 for AS-fixed brains. However, these levels of auto-fluorescence are still reasonable and did not hamper the visibility or the assessment of the antigens, which are still readily apparent. Finally, the IF and IHC techniques were comparable regarding the tissue quality, since the same artifacts (i.e., broken neuropil and shrunken/shriveled cells) were present in slices treated with both visualization techniques, although visible only using DIC when using IF-stained slices.

### Study limits

Our work is not without limitations. First, when assessing the ease of manipulation of the slices, we did not quantify the number of slices that were torn and discarded. Second, although we targeted antigens present in different cells of the central nervous tissue, we did not test many other antigens that could be of interest in neuroscientific research, such as oligodendrocyte markers, cluster of differentiation (CD) markers, extracellular matrix markers, and other myelin antigens (e.g., myelin-associated glycoprotein or myelin oligodendrocyte glycoprotein) (Lyck et al., 2008). Also, we are aware that our analysis was strictly qualitative. We did not consider in-depth quantitative analyses (e.g., stereology to quantify the number of cells, standardized image software to score the backgrounds) at this stage, as we first wanted to assess the feasibility of these procedures (and therefore, their quality) before developing image and cellular counting strategies. Finally, we could also have assessed other basic colorations (e.g., luxol fast blue or cresyl violet), but we considered the assessment of the degradation of specific antigens as a more relevant objective given their use in quantitative neuroscientific research and the impact of chemicals on antigen conformation (i.e., cross-linking) (Puchtler and Meloan, 1985; Aoki et al., 1999; Eltoum et al., 2001; Lyck et al., 2008; Yamaguchi and Shen, 2013; Kuhlmann et al., 2017; Zhou et al., 2017).

Finally, another limitation consists on the comparability of the mouse and the human fixation techniques. The perfusion of the mice is done using gravity and without post-mortem delay, while humans are perfused after variable post-mortem delays and using a pump, which could potentially produce differences in the efficiency of the fixation between the two specimens. However, it is not possible to perfuse full human bodies using a gravity technique, nor it is meaningful to assess fixation by perfusion of dead mice with delays of up to 48 h (potential maximum delay in human anatomy laboratories). Furthermore, mice brains are cryoprotected and frozen following the perfusion, while human brains are extracted after a more lengthy fixation period (up to 8 months) and kept in an aldehyde or alcohol solution until use. However, the goal of the present study was not to assess differences across species, nor to recreate in mice the confounding post-mortem variables that affect human bodies. Our goal was to assess how fixative solutions with different chemical compositions affect brain tissue obtained in optimal conditions, as it is the case with mice. In other words, we designed the study to have a single independent variable (type of solution used) determining the differences in histology procedures and quality (dependent variables). Our results are promising for further studies on human brain tissue, and we acknowledge that the present IHC results could be superior to the results that we will obtain in humans, where the confounding variables related to delayed fixation (among others) cannot be avoided.

### Relevance for human tissue analysis

Even though there are technical and procedural differences in the fixation of mice and human tissue that add confounding variables to the latter (presented in the Study Limits section), there are some similarities in the techniques. For example, we cut blocks of human brains of similar volume than that of mice brains and then apply an identical cryopreservation procedure (overnight immersion in a sucrose solution). Once the tissue is frozen, both mice and human samples are cut in slices of identical thickness using a cryostat. Hence, regardless of the confounding variables, we expect that brain samples will provide useful tissue using IHC stains. Furthermore, we foresee adequate IHC results, given the positive immunofluorescence signal for myelin and neurons that our group obtained in a pilot study using human brains fixed with a salt-saturated solution (Maranzano et al., 2020). Future studies, in human brains fixed with all three different solutions, will address the following: 1—the analysis of cryopreserved and paraffin-imbedded samples, given the relevance of the latter in human histopathology, 2—IHC quality of all antigens, and antigen retrieval techniques, which might be necessary if antigen structures are affected by confounding variables (e.g., long post-mortem delays may induce changes in protein tertiary conformation), and 3—weighting the impact of the confounding variables when collecting enough number of specimens (e.g., comparison of antigenicity in brains fixed 12 h post-mortem vs. 24 h, vs. 36 h, vs. 48 h post-mortem). These studies, which imply much longer periods to collect enough human brain specimens, will fully unveil the real potential of using human brains fixed in gross anatomy laboratories.

## Conclusion

Our work is the first to compare the quality and antigenicity of mice brain tissue fixed with solutions currently used in human gross anatomy laboratories. Even if FAS, routinely used in brain banks and animal fixation, produces better histology quality, we have successfully shown that antigenicity of four cell populations is preserved with two solutions used by anatomists. This opens the door to the use of human brains fixed with SSS and AS to assess different diseases using IHC. Further studies will test the same variables in human brain samples, optimizing the IHC protocol of SSS specimens to reduce the darkness of the background. These studies will also consider the post-mortem delay as a confounding variable, to evaluate the most suitable fixation method for both human dissection and research.

## Data availability statement

The raw data supporting the conclusions of this article will be made available by the authors, without undue reservation.

## Ethics statement

This animal study was reviewed and approved by the Comité de bons soins aux animaux de l’Université du Québec à Trois-Rivières.

## Author contributions

DB and JM contributed to the conception and design of the study. E-MF performed the experimental protocols, organized the database, and performed the statistical analysis. E-MF and JM wrote the manuscript. DB wrote sections of the manuscript. All authors contributed to manuscript revision, read, and approved the submitted version.

## Footnotes

1
https://www.cbc.ca/news/science/brain-banks-crucial-for-research-clamouring-for-donors-1.757008

2
https://www.afmc.ca/en/faculties

3
https://www.cellsignal.com/learn-and-support/protocols/protocol-if-methanol

4
https://www.novusbio.com/support/fixation-and-permeabilization-in-icc-if
